# Supplementary material for: PFN2a Suppresses C2C12 Myogenic Development by Inhibiting Proliferation and Promoting Apoptosis via the p53 Pathway
Source: Cells. 2019 Aug 23;8(9):959. doi: 10.3390/cells8090959 (PMC6770762; doi:10.3390/cells8090959)
Supplement: Supplementary file 1 [file cells-08-00959-s001.pdf]

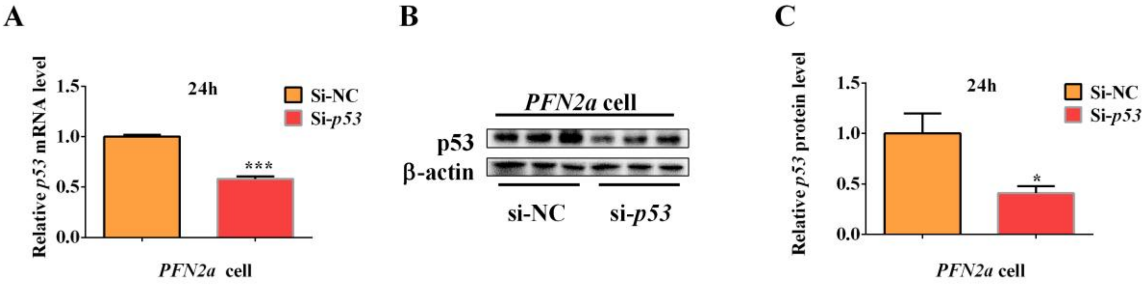

Supplementary Figure S1. si-*p53* decreased the p53 expression on *PFN2a* cell proliferation day 1. (**A**) si-*p53* decreased the mRNA level of *p53* on *PFN2a* cell proliferation day 1. (**B**) si-*p53* reduced the protein level of p53 on *PFN2a* cell proliferation day 1. (**C**) WB band gray scanning results showed si-*p53* reduced the protein level of p53 ($p < 0.05$) on *PFN2a* cell proliferation day 1. The results were presented as mean ± S.E.M. of triplicate experiments for each group, and the statistical significance of differences between means was assessed using unpaired Student's *t*-test (*, $p < 0.05$; ***, $p < 0.001$). *PFN2a* cell: *PFN2a*-overexpressing C2C12 cells; 24h: on *PFN2a* cell proliferation day 1; Si-NC: siRNA-negative control; Si-*p53*: siRNA-*p53*.

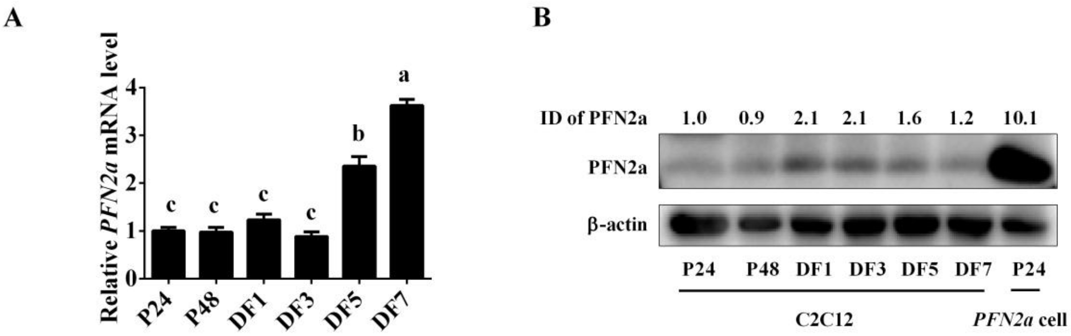

Supplementary Figure S2. *PFN2a* has low mRNA level and protein level during C2C12 myogenic differentiation. (**A**) qPCR analysis of the mRNA of *PFN2a* on C2C12 proliferation at 24 h, 48 h, and on differentiation days 1, 3, 5, and 7, respectively. *PFN2a* was increased on the late stage of C2C12 myogenic differentiation. (**B**) Western blot analysis of PFN2a during C2C12 myogenic development. WB bands gray scanning results showed PFN2a was increased on differentiation days 1 and day 3 compared to proliferation phases. PFN2a was decreased on differentiation day 5 and day 7 compared to the early stages of differentiation. Band intensities were quantified by Image J software and normalized to β-actin. Data were expressed as change in fold relative to the control. The results were presented as mean ± S.E.M. of triplicate experiments for each group, and the statistical significance of differences between means was assessed using one-way ANOVA (SPSS v18.0, IBM Knowledge Center, Chicago, IL, USA). $p < 0.05$ was considered statistically significant. *PFN2a* cell: *PFN2a*-overexpressing C2C12 cells; P: proliferation. DF: differentiation. ID: integrated density.
